# The Bruton's tyrosine kinase inhibitor ibrutinib exerts immunomodulatory effects through regulation of tumor-infiltrating macrophages

**DOI:** 10.18632/oncotarget.16836

**Published:** 2017-04-05

**Authors:** Lingyan Ping, Ning Ding, Yunfei Shi, Lixia Feng, Jiao Li, Yalu Liu, Yufu Lin, Cunzhen Shi, Xing Wang, Zhengying Pan, Yuqin Song, Jun Zhu

**Affiliations:** ^1^ Key Laboratory of Carcinogenesis and Translational Research (Ministry of Education), Department of Lymphoma, Peking University Cancer Hospital & Institute, Beijing 100142, China; ^2^ Key Laboratory of Carcinogenesis and Translational Research (Ministry of Education), Department of Pathology, Peking University Cancer Hospital & Institute, Beijing 100142, China; ^3^ Key Laboratory of Chemical Genomics, School of Chemical Biology and Biotechnology, Peking University Shenzhen Graduate School, Shenzhen University Town, Xili, Shenzhen 518055, China

**Keywords:** ibrutinib, Btk, immunomodulatory effect, macrophages

## Abstract

The Bruton's tyrosine kinase (Btk) inhibitor ibrutinib has demonstrated promising efficacy in a variety of hematologic malignancies. However, the precise mechanism of action of the drug remains to be fully elucidated. Tumor-infiltrating macrophages presented in the tumor microenvironment have been shown to promote development and progression of B-cell lymphomas through crosstalk mediated by secreted cytokines and chemokines. Because Btk has been implicated in Toll-like receptor (TLR) signaling pathways that regulate macrophage activation and production of proinflammatory cytokines, we investigated the immunomodulatory effects of Btk inhibitor on macrophages. Our results demonstrate that Btk inhibition efficiently suppresses production of CXCL12, CXCL13, CCL19, and VEGF by macrophages. Furthermore, attenuated secretion of homeostatic chemokines from Btk inhibitor-treated macrophages significantly compromise adhesion, invasion, and migration of lymphoid malignant cells and even those not driven by Btk expression. The supernatants from Btk inhibitor-treated macrophages also impair the ability of endothelial cells to undergo angiogenic tube formation. Mechanistic analysis revealed that Btk inhibitors treatment downregulates secretion of homeostatic chemokines and cytokines through inactivation of Btk signaling and the downstream transcription factors, NF-κB, STAT3, and AP-1. Taken together, these results suggest that the encouraging therapeutic efficacy of Btk inhibitor may be due to both direct cytotoxic effects on malignant B cells and immunomodulatory effects on macrophages present in the tumor microenvironment. This novel mechanism of action suggests that, in addition to B-cell lymphomas, Btk inhibitor may also have therapeutic value in lymphatic malignancies and solid tumors lacking Btk expression.

## INTRODUCTION

B-cell lymphomas account for approximately 80% of all Non-Hodgkin's Lymphomas and encompass a variety of disease entities with distinct pathological characteristics, clinical features, and prognoses [1]. Gene profiling analysis has revealed that, in addition to neoplastic B cells, non-neoplastic immune cells present in the tumor microenvironment (TME) play an essential role in the biology of lymphoma and can serve as powerful predictors of disease outcomes [2]. The TME is comprised of many cell types, including macrophages, lymphocytes, stromal cells, fibroblasts, and the cells that make up the intratumoral vasculature. These cells promote development and progression of B-cell lymphomas through secretion of cytokines and chemokines that mediate crosstalk between malignant cells and immune cells [3]. For example, tumor-associated macrophages (TAMs) have been shown to secrete the homeostatic chemokines, CXCL12 and CXCL13, which bind to their respective G protein-coupled receptors on malignant B cells, CXCR4 and CXCR5. These interactions promote prosurvival signaling and protect malignant B cells from the cytotoxic effects of chemotherapies [4, 5]. In addition, Macrophage Inflammatory Protein-3 beta (MIP-3β), also called CCL19 is essential for the migration and spreading of mantle cell lymphoma [6]. Thus, TAMs presented in the TME represent a potential therapeutic target for treatment of B-cell lymphoma.

Bruton's tyrosine kinase (Btk) is a member of the Tec family of cytoplasmic tyrosine kinases, which are predominantly expressed in hematopoietic cells [7]. In addition to its essential regulatory role in B-cell proliferation and survival, Btk has also been implicated in promotion of Toll-like receptor (TLR) signaling [8], which regulates macrophage activation and production of proinflammatory cytokines. Several studies have demonstrated crosstalk between Btk and TLR signaling pathways to mediate transactivation of downstream cascades [9, 10]. Furthermore, macrophages from X-linked immunodeficient (xid) mice lacking Btk function exhibit impaired nitric oxide, TNF-α, and IL-1β production, suggesting that Btk plays a critical role in regulation of immunity [11, 12].

Ibrutinib, the first irreversible, small-molecule Btk inhibitor, has demonstrated promising therapeutic effects in patients with B-cell lymphoma [13, 14]. The encouraging clinical efficacy of ibrutinib has been reported to result not only from direct cytotoxic effects on malignant B cells, but also from anti-cancer mechanisms that diminish the contributions of the surrounding microenvironment to cancer cell growth and survival [15–17]. A majority of chronic lymphocytic leukemia and mantle cell lymphoma patients treated with ibrutinib display a transient increase in circulating lymphocytes and reduced lymphadenopathy. These observations are indicative of downregulation of BCR signaling in tumor cells, which leads to mobilization of malignant B cells from the tumor tissue to the peripheral bloodstream. Recently new generations of irreversible Btk inhibitors have exhibited different selectivity profile and more potent anti-tumor activity [18, 19]. Our developed inhibitor PLS-123 presents the dual-action inhibitory mode targeting both Btk catalytic activity and its own auto-activation [20, 21]. However, the precise mechanisms underlying effects of Btk inhibition on macrophages present in the TME are not well understood.

In this study, we report that the Btk inhibitors ibrutinib and PLS-123 exerted immunomodulatory effects through regulation of tumor-infiltrating macrophages and suppressed production of homeostatic chemokines and angiogenic cytokines. Thus, the promising therapeutic effects of Btk inhibition can be attributed to both direct inhibition of BCR signaling in lymphoma cells and immunomodulation of macrophages present in the TME.

## RESULTS

### Btk inhibition suppresses production of homeostatic chemokines and angiogenic cytokines by macrophages

**Figure 1:**
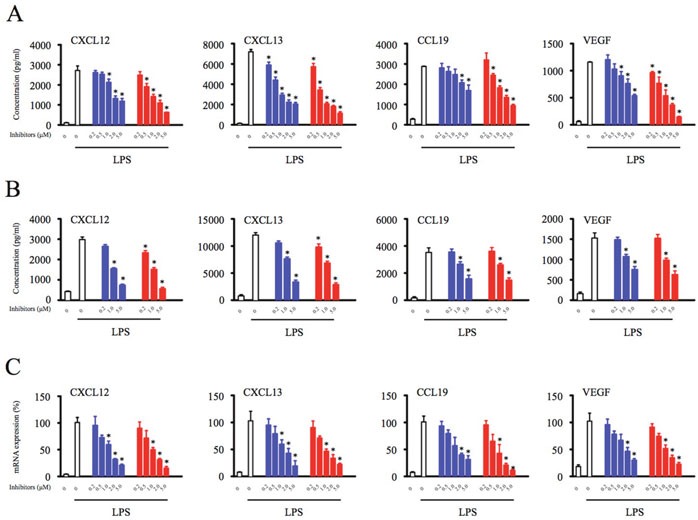
Btk inhibition suppresses production of homeostatic chemokines and angiogenic cytokines by macrophages **(A)** THP-1 differentiated macrophages (2.5 × 10^5^) were pretreated with indicated concentration of Btk inhibitors (0.2, 0.5, 1, 2, 5 μM) for 1 hour. **(B)** PBMCs (1 × 10^6^) were pretreated with indicated concentration of Btk inhibitors (0.2, 1, 5 μM) for 1 hour. After Btk inhibitor washout, the cells were stimulated with LPS (1 μg/ml) for 17 hours and the culture supernatant was harvested for the analysis of the chemokines and cytokine production. **(C)** Total RNA was extracted, and the expression of mRNA was detected by real time PCR. *Significantly decreased compared to LPS treatment alone (*p* < 0.05). Results are representative of three similar experiments.

To characterize the immunomodulatory effects of Btk inhibitors, THP-1 differentiated macrophages were pretreated with the Btk inhibitors ibrutinib and PLS-123 for 1 hour. After Btk inhibitor washout, the supernatants from lipopolysaccharide (LPS)-stimulated cells were analyzed by enzyme-linked immunosorbent assay (ELISA). Importantly, the Btk inhibitors ibrutinib and PLS-123 did not induce cytotoxic effects towards macrophages (Supplementary Figure 1). As shown in Figure 1A, Btk inhibition efficiently downregulated secretion of homeostatic chemokines (CXCL12, CXCL13, and CCL19), as well as the angiogenic cytokine, VEGF, in THP-1 differentiated macrophages. The similar results were also observed in human peripheral blood mononuclear cells (PBMCs; Figure 1B). To further determine whether downregulation occurred at the transcriptional level, total RNA was extracted from Btk inhibitor-treated THP-1 differentiated macrophages, and mRNA expression was analyzed by real-time polymerase chain reaction (PCR). Btk inhibition strongly reduced expression of the CXCL12, CXCL13, CCL19, and VEGF mRNAs, suggesting that Btk activation was required for transcription of these chemokines and this cytokine (Figure 1C).

### Depletion of Btk inhibits macrophage production of homeostatic chemokines and angiogenic cytokines

**Figure 2:**
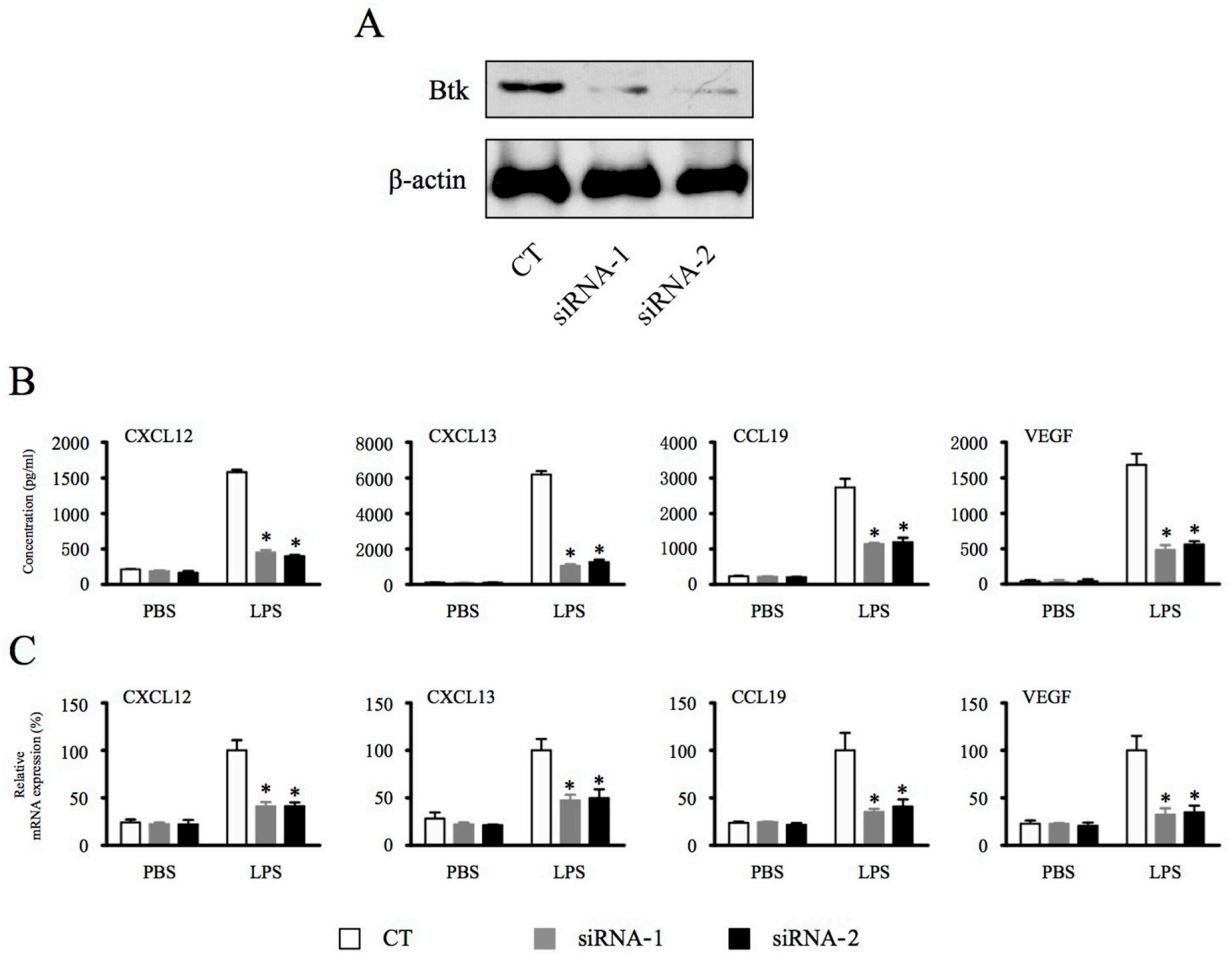
Depletion of Btk inhibits macrophage production of homeostatic chemokines and angiogenic cytokines **(A** & **B)** THP-1 differentiated macrophages were transfected with Btk siRNA for 48 hours and then stimulated with LPS (1 μg/ml) for 18 hours. The chemokine and cytokine production from macrophages were measured by ELISA. **(C)** Total RNA was extracted, and the expression of mRNA was detected by real time PCR. *Significantly decreased compared to LPS treatment alone (*p* < 0.05). Results are representative of three similar experiments.

To further explore the role of Btk in macrophage signaling, the effects of Btk knockdown on chemokine and cytokine production was analyzed. Efficacy of siRNA-mediated knockdown of Btk was analyzed by Western blotting (Figure 2A). Macrophages transfected with Btk-specific siRNAs were stimulated with LPS for 18 hours [21], and levels of chemokines and cytokine in the supernatant were measured by ELISA. Btk knockdown significantly inhibited secretion of CXCL12, CXCL13, CCL19, and VEGF by macrophages (Figure 2B). Similarly, PCR analysis of siRNA-transfected macrophages demonstrated that loss of Btk expression blocked expression of homeostatic chemokines and the angiogenic cytokine at the transcriptional level (Figure 2C).

### Inhibition of Btk function in macrophages significantly compromises adhesion, invasion, and migration of lymphoid malignant cells

**Figure 3:**
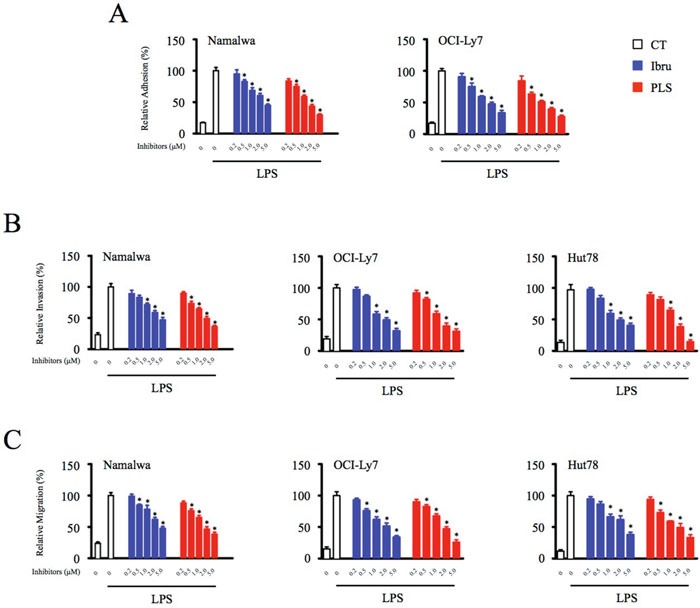
Inhibition of Btk function in macrophages significantly compromises adhesion, invasion, and migration of lymphoid malignant cells **(A)** Lymphoid malignant cells were subjected to adhesion assays in conditioned medium collected from control and Btk inhibitor-treated (0.2, 0.5, 1, 2, 5 μM) macrophages. The adherent cells were measured by CellTiter-Glo luminescent cell viability assay. **(B** & **C)** Migration and invasion of tumor cells were analyzed in transwell plates, and supernatant from macrophages was added into the lower chamber as a chemoattractant. *Significantly decreased compared to LPS treatment alone (*p* < 0.05). Results are representative of three similar experiments.

Tumor-infiltrated macrophages have been shown to promote progression of B-cell lymphomas through intercellular crosstalk mediated by cytokines and chemokines [3]. In addition, ibrutinib treatment has been demonstrated to efficiently inhibit adhesion and migration of malignant B cells through downregulation of chemokine-mediated Btk activation within tumor cells [17]. To investigate whether immunomodulatory effects of Btk inhibition on macrophages present in the TME affect tumor cell function, malignant B-cell lymphoma Namalwa and OCI-Ly7 cells were co-cultured with supernatants collected from control and Btk inhibitors-treated macrophages. Consistent with results showing that Btk inhibition decreases chemokines and cytokine production (Figure 1), adhesion of malignant B cells to fibronectin was attenuated by supernatant exposure in a dose-dependent manner (Figure 3A).

To further assess the impact of Btk inhibition of macrophages on tumor cell function, supernatant-treated Namalwa and OCI-Ly7 cells were subjected to an *in vitro* motility assay using a transwell culture system. Btk inhibitor-mediated decreases in chemokine and cytokine levels in macrophage supernatants were associated with concomitant decreases in the ability of malignant B cells treated with these supernatants to undergo invasion and migration. Even more importantly, the supernatants collected from Btk inhibitors-treated macrophages also strongly diminished the motility of T-cell lymphoma Hut78 cells, suggesting immunomodulatory effects of Btk inhibition on macrophages presented in the tumor microenvironment may also have therapeutic value in lymphatic malignancies not driven by Btk expression (Figure 3B and 3C; Supplementary Figure 2). Thus, in addition to their direct effects on tumor cells, Btk inhibitors likely also indirectly blocked the motility of neoplastic cells through downregulation of chemokine and cytokine production by macrophages.

### Btk inhibition of macrophages affects endothelial cell tube formation

**Figure 4:**
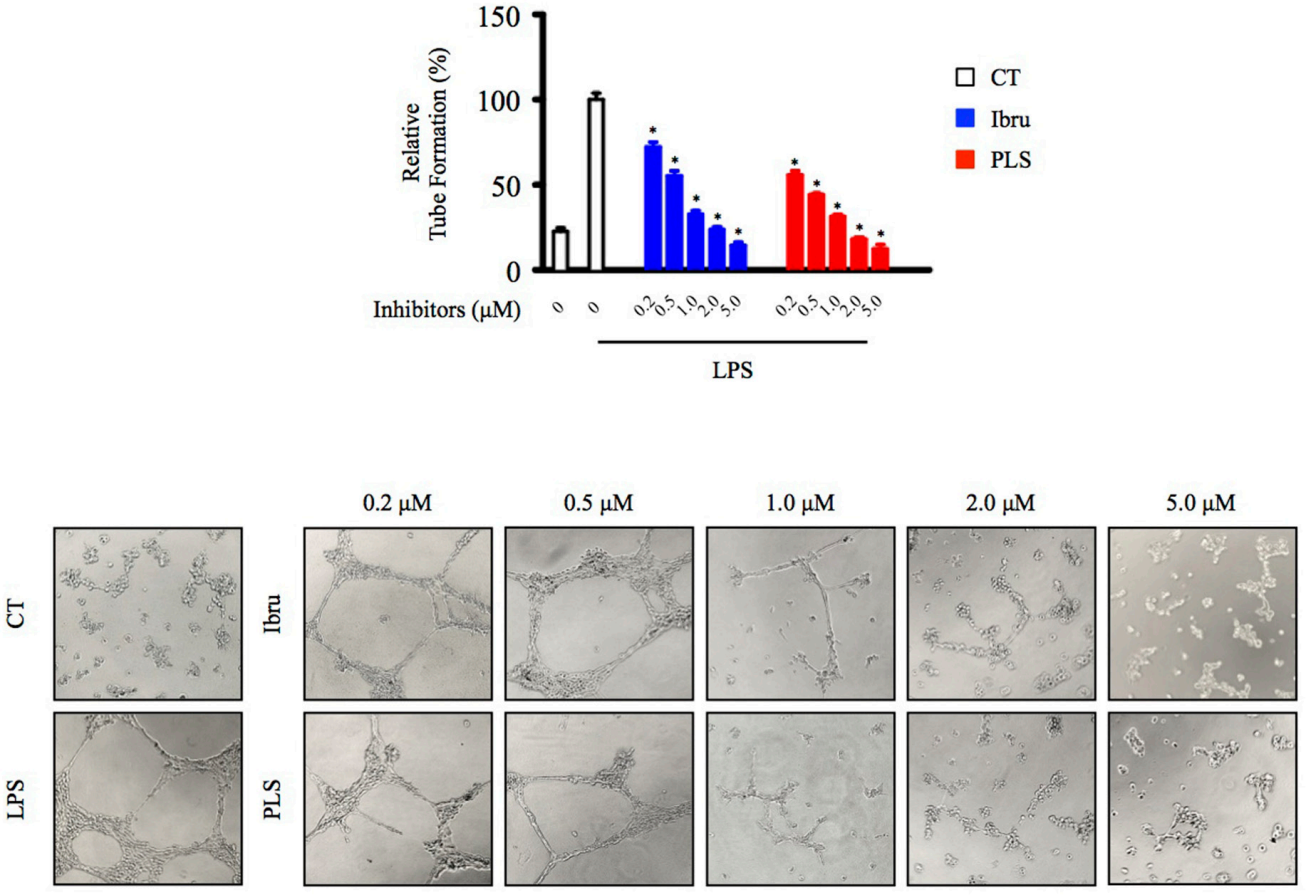
Btk inhibition of macrophages affects endothelial cell tube formation HUVEC cells were seeded into a Matrigel-coated plate and incubated with conditioned medium from macrophages for 20 hours. Tube-like structures of HUVECs formed were counted under a microscope. *Significantly decreased compared to LPS treatment alone (*p* < 0.05). Results are representative of three similar experiments.

New growth of the vascular network plays an important role in progression of lymphoma and is dependent upon communication between various cell types present in the TME [22]. Thus, we hypothesized that treatment of macrophages with Btk inhibitors would compromise VEGF production and angiogenesis in the TME. A Matrigel assay revealed that treatment of endothelial cells with supernatants from control macrophages induced endothelial cell tube formation, while treatment with supernatants from Btk inhibitor-treated macrophages reduced tube formation in a dose-dependent manner (Figure 4). Thus, these results suggested that Btk may exert affects on angiogenesis through modulation of cytokine production by TAMs.

### Btk inhibition blocks activation of Btk and downstream signaling cascades

**Figure 5:**
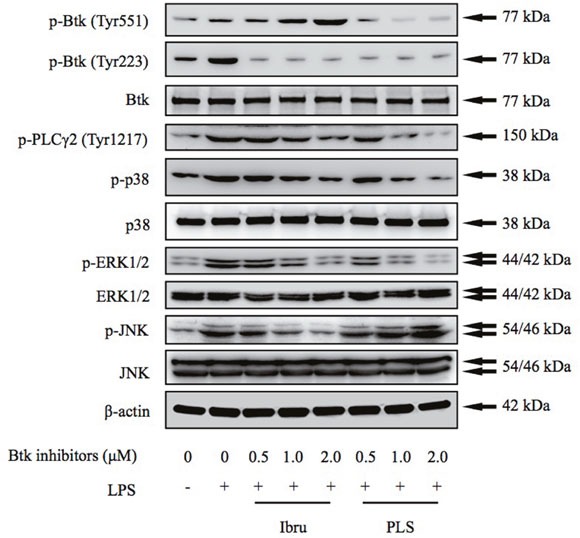
Btk inhibition blocks activation of Btk and downstream signaling cascades THP-1 differentiated macrophages were pretreated with indicated concentration of Btk inhibitors for 1 hour and then stimulated or not with LPS (1 μg/ml) for 10 minutes. Whole cell extracts were probed by Western blot for Btk, PLCγ2, ERK, p38, and JNK. β-actin is shown as a loading control. Results are representative of three similar experiments.

To investigate the mechanisms by which Btk inhibition blocks production of homeostatic chemokines and an angiogenic cytokine by macrophages, THP-1 differentiated macrophages were incubated with Btk inhibitors in the presence or absence of LPS stimulation. LPS stimulation is thought to mimic TLR/antigen interactions, thereby activating downstream signaling pathways [23]. Results from this analysis revealed that ibrutinib inhibited the LPS-induced Btk Tyr223 phosphorylation and the negative pathway feedback loop, resulting in amplified Tyr551 phosphorylation [24]. The dual-action Btk inhibitor PLS-123 not only significantly suppressed Btk Tyr223 phosphorylation but also reduced elevated Btk Tyr551 phosphorylation. Furthermore, Western blot analysis also demonstrated that Btk inhibition efficiently reduced downstream activation of PLCγ2 and MAPK family proteins (p38, ERK1/2 and JNK) [25], consistent with results showing that Btk inhibition downregulates chemokine and cytokine production. Interestingly, the phosphorylation of JNK was slightly upregulated by PLS-123 treatment (Figure 5).

### Btk regulates homeostatic chemokine and angiogenic cytokine production through activation of NF-κB, STAT3, and AP-1

**Figure 6:**
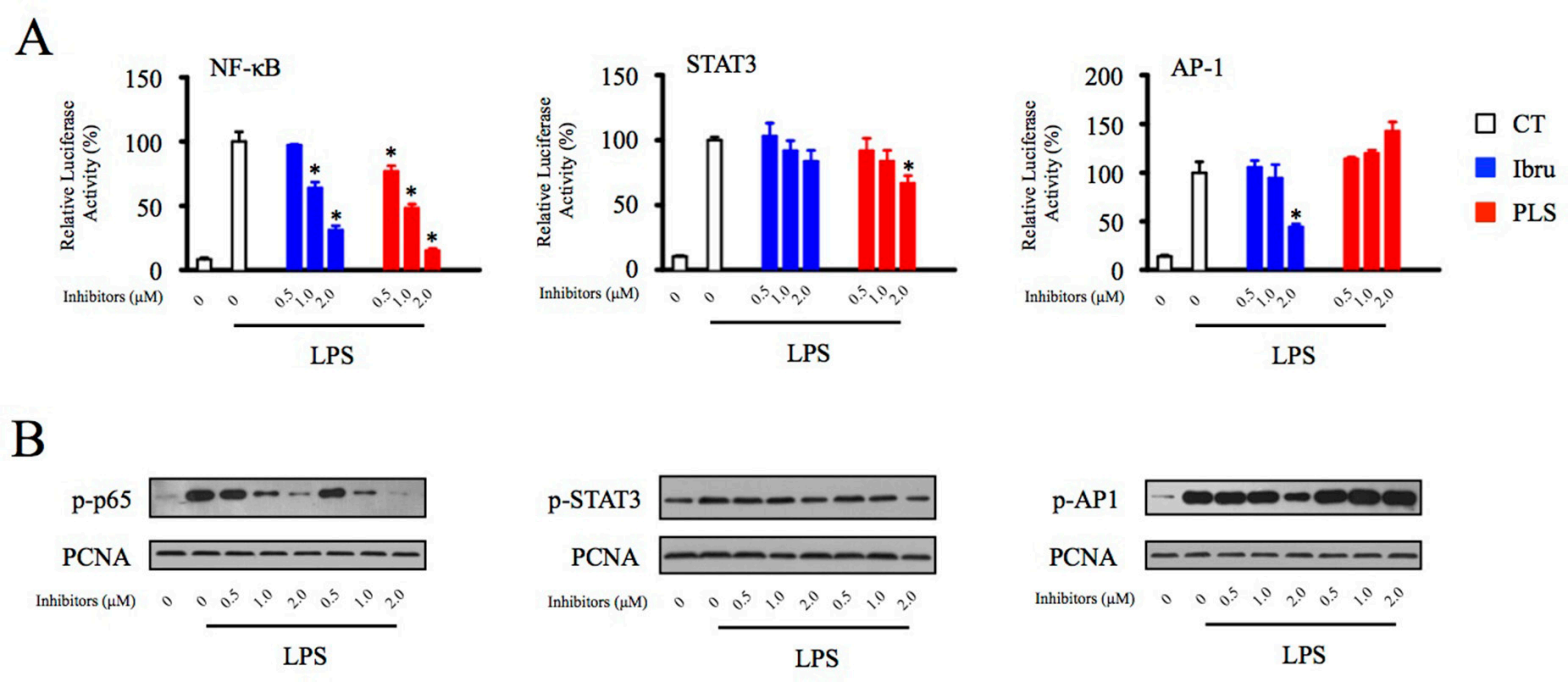
Btk regulates homeostatic chemokine and angiogenic cytokine production through activation of NF-κB, STAT3, and AP-1 **(A)** THP-1 differentiated macrophages were transfected with NF-κB, STAT3 and AP-1 luciferase reporter constructs and then treated with indicated concentration of Btk inhibitor in the presence or absence of LPS stimulation (1 μg/ml) for 6 hours prior to the measurement of luciferase. **(B)** THP-1 differentiated macrophages were pretreated with the indicated concentrations of Btk inhibitors for 1 hour and then stimulated or not with LPS (1 μg/ml) for 10 minutes. The nuclear extracts of cultured cells were prepared and probed by Western blot analysis to measure p-p65, p-STAT3 and p-AP1 levels. PCNA is shown as a loading control. *Significantly decreased compared to LPS treatment alone (*p* < 0.05). Results are representative of three similar experiments.

Because transcription factors are key regulators of chemokine and cytokine production, we next analyzed the effects of Btk inhibition on activation of NF-κB, STAT3, and AP-1 using a luciferase reporter assay in macrophages. Treatment with LPS resulted in significant increase in reporter activities associated with all three transcription factors. Reporter activity of NF-κB and AP-1 was strongly reduced by incubation with ibrutinib. The dual-action Btk inhibitor PLS-123 decreased the NF-κB and STAT3 activation. Similar to results from the reporter assay, the activation of transcription factor was also confirmed by Western blot analysis of nuclear extracts prepared from control and Btk inhibitor-treated macrophages (Figure 6). Consistent with previous immunoblotting result of JNK phosphorylation, the nuclear transcription factors AP-1 activation was slightly increased by PLS-123 treatment, which suggested its alternative mechanism of immunomodulatory effect towards macrophages. Together, these results suggested that Btk inhibition exerts anti-tumor immunomodulatory effects through the regulation of NF-κB, STAT3, and AP-1 activation in macrophages.

## DISCUSSION

Ibrutinib, the first selective, irreversible, small-molecule inhibitor of Btk, covalently binds with cysteine 481 (Cys-481) near the active site of the kinase to block its enzymatic activity [26]. Ibrutinib has clearly demonstrated promising therapeutic effects in clinical trials and has been approved by the Federal Drug Administration (FDA) for treatment of chronic lymphocytic leukemia, mantle cell lymphoma, and Waldenström's macroglobulinemia. Importantly, the encouraging clinical efficacy of ibrutinib results from both direct cytotoxic effects on cancer cells, including activation of apoptosis, inhibition of DNA replication, and blockade of prosurvival signaling pathways, as well as from inhibition of interactions between tumor cells and the surrounding microenvironment that affect cellular adhesion and migration [27]. Recently, the immunomodulatory effect of ibrutinib has been reported to enhance the anti-tumor immune response of infiltrating T cells and reduce chronic graft-versus-host disease [28, 29]. Thus, these observations suggest that the effects of Btk inhibition on the TME require more detailed investigation.

Many lines of evidence indicate that the frequency and distribution of non-neoplastic immune cells in the TME is strongly associated with the outcome and prognosis of lymphoma [30]. TAMs promote tumor progression through secretion of homeostatic chemokines, pro-angiogenic factors, and suppressive cytokines, which limit the anticancer immune response [31]. Because Btk is expressed in macrophages and its phosphorylation is upregulated upon macrophage activation, we hypothesized that the promising anti-tumor activity of ibrutinib might be partially attributed to immunomodulatory effects on TAMs present in the TME. Understanding the complete mechanism of action of ibrutinib will help us to better explain the clinical findings associated with the drug, as well as identify potential new therapeutic uses.

Chang et al. previously demonstrated that B-cell lymphoma patients treated with ibrutinib display reduced lymphadenopathy, accompanied by transient increases in circulating malignant B cells in the peripheral bloodstream [17]. The underlying mechanism for this transient increase has been attributed to ibrutinib-mediated inhibition of chemokine-mediated adhesion and migration resulting from decreased stimulation of phosphorylated Btk (p-Btk) and phosphorylated PLC-γ2 (p-PLCγ2) by CXC12/CXCL13 in malignant B cells. However, results from our present study reveal that Btk inhibitors also exert immunomodulatory effects on macrophages that lead to decreased chemokines and cytokine production. Furthermore, supernatants collected from Btk inhibitors-treated macrophages efficiently compromise the adhesion, invasion and migration function in lymphoid malignancies and even those not driven by Btk expression. Thus, attenuated CXCL12, CXCL13, and CCL19 levels in the TME may limit the interactions between malignant cells and surrounding non-neoplastic immune cells, thereby contributing to mobilization of lymphoid malignant cells from tumor tissues to the peripheral bloodstream through a cancer cell-independent mechanism. This novel mechanism of action suggests that, in addition to B-cell lymphomas, Btk inhibitors may also displayed impressive therapeutic potency in lymphatic malignancies and solid tumors lacking Btk expression.

Tec family kinases, such as Btk, have been shown to activate downstream signaling pathways and transcription factors [32, 33]. Activation of the transcription factors NF-κB p65, STAT3, and AP-1 induces expression of genes responsible for inflammation, including CXCL12, CXCL13, CCL19, and VEGF, which play important roles in the TME [34–37]. Therefore, we investigated the transcriptional mechanisms underlying Btk inhibitor-mediated decreases in chemokine and cytokine production. Data from pharmacological studies and Western blot assays demonstrated that Btk inhibition reduced chemokine production through downregulation of Btk phosphorylation and activation of downstream transcription factors (Figures 5 and 6). These findings suggest that targeting Btk activation in TAMs could be an efficient therapeutic strategy that exerts anti-cancer effects through impacts on the TME.

Increasing evidences indicate that targeting TAMs present in the TME significantly reduces tumor progression and improves the efficacy of chemotherapy [38]. For example, the chemotherapeutic agent trabectedin exhibited remarkable anti-tumor activity resulting from cytotoxicity towards mononuclear phagocytes [39]. CSF-1R inhibitors represent a promising therapeutic approach for targeting TAMs. CSF-1/CSF-1R signaling has been shown to play essential roles in regulating differentiation and survival of macrophages and driving macrophage recruitment into tumor tissues [40, 41]. Results from a phase I clinical trial demonstrated that the CSF-1R-targeted monoclonal antibody RG7155 depleted CSF-1R^+^ CD163^+^ macrophages in tumor tissues and had significant clinical benefit in patients with diffuse-type giant cell tumors. Thus, chemo-immunomodulatory strategies targeting macrophages can successfully limit tumor progression and metastasis.

In conclusion, our results suggest that Btk inhibition modulates immune responses in the TME. These previously unrecognized immunomodulatory mechanisms associated with Btk inhibitors indicate that evaluation of this drug in other lymphatic malignancies and solid tumors, even those lacking Btk expression, may be warranted.

## MATERIALS AND METHODS

### Cell lines and culture conditions

The human acute monocytic leukemia cell line THP-1 and B-cell lymphoma cell line Namalwa and OCI-Ly7 were kindly provided by Dr. Fu (University of Nebraska Medical Center, Omaha, NE). The cutaneous T cell lymphoma cell line Hut78 were obtained from ATCC (Manassas, VA). All cells were grown in DMEM supplemented with 10% fetal bovine serum (FBS; Gibco, Life Technology, New Zealand), L-glutamine and penicillin-streptomycin in a humidified atmosphere of 5% CO_2_ at 37°C. The authentication of all cell lines was performed using short tandem repeat DNA fingerprinting analysis (Applied Biosystems, Foster City, CA). THP-1 monocytes were treated with 50 ng/ml of phorbol-12-myristate-13-acetate (PMA; Sigma-Aldrich, St. Louis, MO) for 48 hours to induce macrophage differentiation [42]. For conditioned medium generation, supernatants from THP-1 differentiated macrophages were collected, sterilized through a 0.22 μm filter, and kept at 4°C for further use. Human peripheral blood mononuclear cells (PBMCs) were freshly prepared from 20 ml of EDTA-blood from healthy volunteers. Cells were isolated using Lymphoprep (Axis Shield, Oslo, Norway) according to the protocol from the manufacture.

### Reagents and antibodies

The Btk inhibitors ibrutinib and PLS-123 were synthesized at the laboratory of Dr. Zhengying Pan at Peking University Shenzhen Graduate School according to a previously published procedure [21, 26]. Antibodies against phospho-Tyr223-Btk (#5082), Btk (#8547), phospho-Tyr1217-PLCγ2 (#3871), PLCγ2 (#3872), phospho-p38 (#9211), p38 (#8690), phospho-ERK1/2 (#4370), ERK1/2(#9102), phosho-JNK (#9251) and JNK (#9252) were obtained from Cell Signaling Technology (Danvers, MA, USA). The anti-p65 (phospho S536), STAT3 (phospho Y705), AP-1 (phospho S63) and PCNA were purchased from Abcam (Cambridge, MA). The anti-phospho-Tyr551-Btk, β-actin (A5441) and Lipopolysaccharide (LPS) were obtained from BD Biosciences (San Jose, CA) and Sigma.

### ELISA

THP-1 differentiated macrophages were treated with the indicated Btk inhibitors in the presence or absence of LPS stimulation, and supernatants were collected for the analysis of CXCL12, CXCL13, CCL19 and VEGF production by using ELISA kits (PeproTech, London, UK).

### Real-time quantitative PCR

THP-1 differentiated macrophages were treated with Btk inhibitors in the presence or absence of LPS for 12 hours, and total RNA was extracted via RNA TRIzol Extraction (Life Technologies). The mRNA levels of CXCL12, CXCL13, CCL19, and VEGF were determined by SYBR real-time quantitative PCR kit. The primer sequences of the target genes are listed as follow: β-actin, 5′-CCTGGCACCCAGCACAAT-3′, and 5′-ATCAAGATCATTGCTCCTCCTGAGCGC-3′; CCL-12: 5′-TCCAAACTGTGCCCTTCA-3′, and 5′-AC TTTAGCTTCGGGTCAAT-3′, CCL-13: 5′-GCTCAG TCTTTATCCCTA-3′, and 5′-TTCTCAATACTTCCATC A-3′, CCL-19: 5′-GGCACCAATGATGCTGAA-3′, and 5′-CTCTGGATGATGCGTTCTAC-3′, VEGF: 5′-CTG GAGCGTGTACGTTGGT-3′, and 5′-TCGTTTAACTC AAGCTGCCTC-3′.

### siRNA transfection

THP-1 differentiated macrophages were seeded on 24-well plates in complete media on the day of the transfection. siRNA oligonucleotides targeting Btk (1: 5′-GGCAGUAAGAAGGGUUCAATT-3′; 2: 5′-GU GAUCUGGUUCAGAAAUATT-3′) were synthesized by Invitrogen and transfected to cells using the Lipofectamine™ RNAiMAX reagent (Life Technologies). The efficiency of siRNA knockdown of target genes was determined by Western blotting.

### Cell adhesion and motility assay

Lymphoid malignant cells were treated with the conditioned medium derived from macrophages for 3 hours, and then cells were seeded into 96-well plates that were precoated with fibronectin (BD Biosciences). Thirty minutes later cells were washed twice with PBS to remove the non-adherent cells, and adherent cells were measured by CellTiter-Glo luminescent cell viability assay kit. For relative adhesion determination, the group treated with LPS alone was set to be 1 and the other group was normalized to this group.

For cell invasion and migration assay, tumor cells suspended in serum free medium were pre-labeled with calcein-AM (Sigma) for 30 minutes, then cells were plated into FluoroBlok™ inserts (Corning), which were precoated with Fibronectin or not. The inserts were placed in a 24-well plate containing conditioned medium from macrophages. Twelve hours later, tumor cells in the lower chambers were measured by plate reader at bottom reading mode and visualized by an inverted fluorescence microscope (TCS-SP2, Leica Microsystems).

### Tube formation assay

Growth factor-reduced Matrigel were distributed in 24-well plates and allowed to solidify at 37°C for at least 1 hour. HUVECs incubated in M199 containing 1% FBS for 6 hours were harvested after trypsin treatment and suspended in macrophage-derived conditioned medium. HUVECs were then seeded onto the solified Matrigel. After 20 hours, the cultures were observed and digital pictures were captured with phase contrast microscopy (Axio Observer A1, Zeiss). For relative tube formating determination, the group treated with LPS alone was set to be 1 and the other group was normalized to this group.

### Western blotting

To prepare whole cell extracts, PMA-differentiated macrophages were lysed with RIPA lysis buffer [21]. Equivalent amounts of protein (10 μg) were resolved on SDS-PAGE gels, transferred and immobilized on nitrocellulose membranes (Amersham, Buckinghamshire, UK) and probed with the appropriate primary and secondary antibodies. Immunodetection was performed using a chemiluminescence detection system (Alpha Innotech, San Leandro, CA).

### Transfection and luciferase reporter assay

THP-1 differentiated macrophages were transfected with the transfection reagent Lipofectamine 2000 Reagent (Invitrogen, Carlsbad, CA) at 3 μl of reagent per microgram of DNA. Briefly, the NF-κB, STAT3 and AP-1 luciferase reporter plasmids were transfected to 90% confluent cells in 24-well culture plates. Eighteen hours after transfection, cells were treated with Btk inhibitor in presence or absence of LPS stimulation. After an additional 6 hours of incubation, cells were lyzed and luciferase activity was measured using Synergy 2 microplate reader (BioTek Instruments, Winooski, VT, USA).

### Statistical analysis

All experiments were repeated more than three times and representative results are shown in the figures. Results are expressed as mean ± SD. Statistical analysis was performed using Student's *t* test. A confidence level of *p* < 0.05 was considered significant.

## SUPPLEMENTARY FIGURES



## Abbreviations

BCR signal: B cell receptor signal
VEGF: vascular endothelial growth factor
JNK: c-Jun N-terminal protein kinase
ERK: extracellular-signal regulated kinases
PCNA: proliferating-cell-nuclear-antigen
NF-κB: nuclear factor ‘kappa-light-chain-enhancer' of activated B-cells
STAT3: signal transducer and activator of transcription 3
AP-1: activator protein 1
Itk: IL-2-inducible tyrosine kinase
CSF-1: colony stimulating factor-1
CSF-1R: CSF-1 receptor
MAPK: mitogen-activated protein kinases
