# Supplementary material for: The Bruton's tyrosine kinase inhibitor ibrutinib exerts immunomodulatory effects through regulation of tumor-infiltrating macrophages
Source: Oncotarget. 2017 Apr 5;8(24):39218–29. doi: 10.18632/oncotarget.16836 (PMC5503608; doi:10.18632/oncotarget.16836)
Supplement: Supplementary file 1 [file oncotarget-08-39218-s001.pdf]

The Bruton's tyrosine kinase inhibitor ibrutinib exerts immunomodulatory effects through regulation of tumor-infiltrating macrophages

SUPPLEMENTARY MATERIALS

SUPPLEMENTARY FIGURES

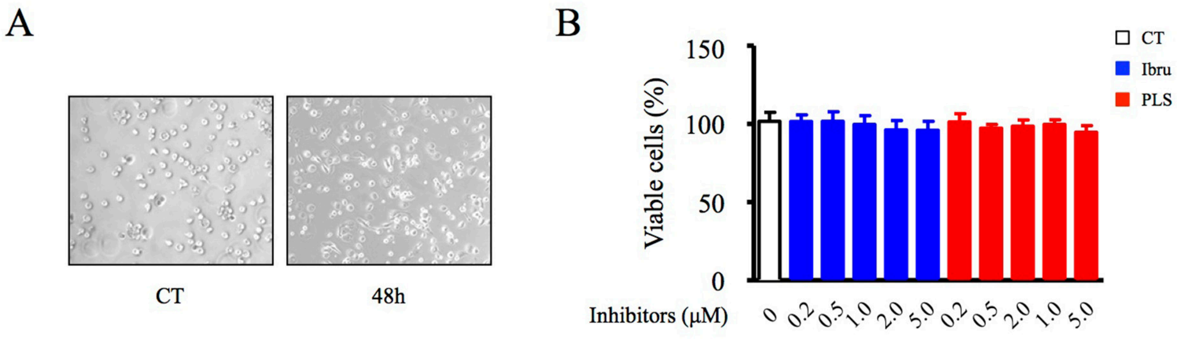

Supplementary Figure 1: Cytotoxicity effect of Btk inhibitors in THP-1 differentiated macrophages. **(A)** THP-1 cells were treated with PMA (50 ng/ml) for 48 hours to induce macrophage differentiation. **(B)** THP-1 differentiated macrophages were incubated with the indicated concentrations of ibrutinib, PLS-123 or vehicle for 18 hours. The cell viability was determined using the Cell Titer-Glo luminescent cell viability assay. Results are representative of three similar experiments.

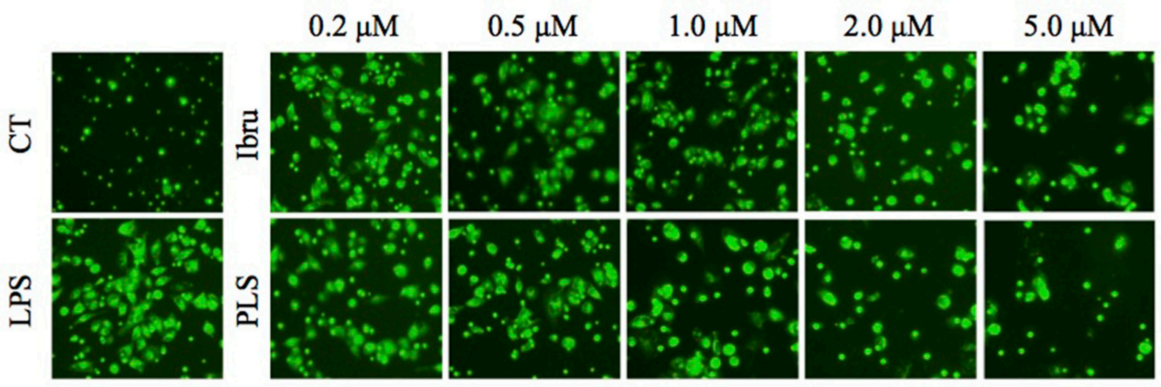

Supplementary Figure 2: Migration and invasion analysis of lymphoid malignant cells. Namalwa cells suspended in serum free medium pre-labeled with calcein-AM were plated into transwell insert chambers, which were precoated with fibronectin or not. The supernatant from macrophages was added into the lower chamber as a chemoattractant. Twelve hours later, Namalwa cells in the lower chambers were visualized by an inverted fluorescence microscope. Results are representative of three similar experiments.
